# Dialog between artificial intelligence & natural intelligence

**DOI:** 10.1002/qub2.5

**Published:** 2023-11-02

**Authors:** Michael Q. Zhang

**Affiliations:** ^1^ Department of Biological Sciences Center for Systems Biology University of Texas Dallas Texas USA

Recently, *Quantitative Biology* (*QB*) held a discussion on “AI (artificial intelligence) for Life Science” among editorial board members and interested scholars in anticipation of rapid development of this growing area after *AlphaGo* and *ChatGPT* mania. Many young people tend to get confused between facts and fictions; heated debates are unavoidable even among their mentors. When deep learning as represented by convolutional neural networks and LSTM (long short‐term memory) was made available for bioinformatics students, many of them rushed into this research field and tried to adopt these methods in all their projects without knowing the history that these tools were becoming successful consistently with Moore’s Law (relating to rapid computer technology advances), but more importantly due to new structural/functional understanding of vision and auditory circuits in the brain. Recently, some young people have claimed “LSTM is dead, long live transformer” (which is somewhat like saying “the bike is dead, long live the car”), and have amplified the threat that *ChatGPT* could wipe out human jobs. They believe transformer is the “silver bullet” for all learning tasks, clearly reflecting their lack of basic knowledge (i.e. “No Free Lunch Theory,” the trade‐off of such global “attention network” is to pay the price for complexity: difficulty of training and high memory costs). There is no doubt ML (machine learning) and AI have brought a new revolution in science and technology, and will deliver huge unforeseeable impact to human everyday life as well as to social relationships. In this context, *QB* journal could be a great platform for encouraging intellectual discussions and for promoting AI for Life Science. Here, I would like to use the DIALOG to “抛砖引玉” (make some initial remarks to get the ball rolling), although it is my personal opinion which is inevitably subject to bias and limitations.

## 1 WHAT IS THE DIFFERENCE BETWEEN AI & NI (NATURAL INTELLIGENCE)?

**AI**: Do you know my name “Artificial Intelligence” is defined by the Oxford English Dictionary as the capacity of computer systems (which may be referred as a “robot”) to exhibit or simulate your intelligent behavior?

**NI**: Wait a minute, intelligence itself is defined as the ability to learn, understand and think in a logical way. Can you think?

**AI**: No. But that definition is too restrictive, actually intelligence has different scopes and degrees. Simple intelligent control devices date back to antiquity, from windmills to thermostat.

**NI**: Agree, everything is relative. Macromolecules (e.g., enzyme) and cells (e.g., immune cell) might be considered to be intelligent; see how a white blood cell is chasing bacteria in the youtube website (search for “Crawling neutrophil chasing a bacterium”). Our emergent/collective intelligent behavior does not require a brain or even a neuron; see how slime molds can solve optimization—Hamilton cycle‐problem more effectively than a human in the youtube website (search for “Intelligence without a brain?”). Before there was any neuron, Ca^2+^ sensing and signaling were already fully functional. Even if one knock‐out a neural circuit, redundant signaling pathways, albeit on much local and slower scale, could still function by themselves (just as if highways were demolished, local roads/paths would still be working). In fact, the most detailed “Neural signal propagation atlas of *Caenorhabditis elegans*” [1] demonstrated that functional connectivity differs from anatomy (connectome) because extra‐synaptic signaling also drives neural dynamics! Worm brain connectomes are largely invariant but every human brain connectome is very different (depending the diversity of learning experience). The human brain functional activity is far more complex than that of a worm brain, certainly beyond what a neural circuit could explain.

**AI**: Well, that’s impressive. I thought only we could beat humans, albeit only in certain specified areas for now. My master promise to make an artificial general intelligent (AGI) robot which can understand or learn any intellectual task that you humans or other animals can.

**NI**: Well, that is not possible and is not an appropriate goal either. It is not possible because we are an evolutionary/developmental product (with a long history of learning and memory from evolutionary tinkering): our living objective goal is survival of the population. You, on the other hand, are an engineering product (efficiently and optimally designed): your goal is to extend and maximize human capability. It makes sense to complement the human brain, but foolish and dangerous to try to replace it.

**AI**: We are not satisfied with merely passing the Turing Test; most of us don’t care if we could really think as long as we could act like we think (that is, as if we do have a mind and consciousness, as expressed in the so called “Weak AI hypothesis”). After all, the brain is a computer; a neural network is just an electric or ionic circuit. Logical computing does not need to be based on living cells.

## 2 COMPLEXITY OF A NEURON AND ORIGIN OF NEURAL NETWORK

**NI**: That is not true, because a neuron is not just a simple node (logic gate) and neural network is not fixed circuit, neither as in Pitts&Mcculloch perceptron model. A single neuron, even a single dendrite, is much more complicated and far more powerful than a full‐blown deep‐learning artificial neural network (ANN) [2].

**AI**: Even though single neurons are complex computational devices (dendritic non‐linear), running an equivalent multilayer ANN is 2000 times faster than computing with biophysics N‐methyl‐D‐aspartate receptor channel models [3]. More information can be found in the youtube website (search for “Dendrites: why biological neurons are deep neural networks”).

**NI**: Often silicon computing (CPU, GPU) is much faster than brain computing (action potential, ms); but there is no comparison in energy efficiency. Bacteria sensing (chemotaxis computation), powered by ATP (adenosine triphosphate) hydrolysis, uses very little energy that is close to the Landauer limit, whereby achieving or maintaining one bit of information requires minimum of 1 kT ln (2) free energy [4]. The human brain consumes oft‐quoted 20 W, compared to the AlphGo system 1 MW! More recent estimate of energy audit is only 0.1 W to cortical computing, and long‐distance communication cost is 3.5 W [5].

## 3 EVOLUTION AND DEVELOPMENT

**AI**: Assuming we have infinite computing resources and an infinite amount of training data, not only could we speak human languages, but we could also derive physical laws, prove mathematics theories, and even re‐engineer the structure and mechanism of brain and carry out any logical computations that are necessary to understand natural laws and human behaviors. It is only a matter of time before we surpass human intelligence, achieving AGI and free will, too!

**NI**: Unfortunately, nothing is infinite and nothing is free either; everything is constrained by physical laws (Planck’s constant sets the finite limit both in the small and in the large) and by evolutionary history (not just of biological living creatures, but also of a “living” galaxy and our universe). Let’s just focus on animal evolution. Most human neural networks do not do logical computations at all; basic survival simply cannot be dependent on reasoning. Indeed, the prefrontal cortex‐the small part of the brain that is key for reasoning, is the last to mature (∼20 years old) in development, only emerged at the root of the evolutionary tree of great apes (∼15 mya) and language appeared even much later. Even for logical inference, NI is focusing more on statistical properties, as von Neumann rightly pointed out, trading arithmetical precision and speed for reliability.

**AI**: My engineers mostly focus on emulating brain, but the CNS (central nerve system) also includes the spinal cord; most of them do not know that in addition to CNS, there are also PNS (peripheral nervous systems) and ENS (enteric nervous systems), right?

**NI**: Yes, they are the keys to why you do not have feelings because you do not have heart and gut! Even if you could pretend to have them (such as in an advanced ChatGPT or humanoid), you could never avoid the uncanny valley phenomenon.

**AI**: Maybe that is at the heart of Moravec’s paradox, namely the dichotomy of intelligence whereby anything easy for a human would be hard for a robot, and vice versa?

**NI**: This is related to the nature and nurture problem; something built‐in (e.g., a baby sucking nipple for milk with feeling and connection to mother) is clearly rather difficult if not impossible for a robot. But the paradox only looks at one side; another side could be more fatal. Although AI may solve more problems and be faster, AI can never propose a good problem/hypothesis (a good problem is not just intellectually changing and interesting, but is also feasible and appropriate).

**AI**: You make me less confident to compete with human instinct or creative intelligence. I can see that even if I had a heart, I would not know what “feeling” I could have; certainly nothing would be comparable or match to those of a human being. When two people watch the same art or movie, one could feel love but another could feel hate! And if a thousand people watch, a wide spectrum of reactions would result, depending on more details such as the different individuals’ specific genes, developments and experiences.

**NI**: Therefore, you cannot and should not try to match to general human intelligence. You cannot because you do not contain the vital memory of billion years of evolution which is encoded in our genes; conversely your assembly cannot compare with natural development so that our phenotype (including morphological forms and behavior maturation) is decoded through multi‐spatial‐temporal scales subject to natural selection at all levels. You should not, because as human extensions or helpers as all engineering products are, you should just do jobs that complement human capacity.

**AI**: In some medical applications we could help to correct human defects or could even replace brain circuit by chips! Humans may not allow us to replace the whole brain though. Medically if a brain is dead, the person is proclaimed dead, although presumably some PNS and ENS should still function in a vegetative state.

**NI**: Even if you could replace the whole brain, the person is no longer the same person, but in fact is not a person at all, but walking dead (行尸走肉). It would take too long to explain that evo‐devo is necessary for NI, and cannot be realized by AI. I suggest reading of Gerald Maurice Edelman (Nobel Laureate in Immunology) books, especially *Bright Air, Brilliant Fire, On the Matter of Mind* (1992). Although not everyone agrees with Neural Edelmanism, anyone who is serious about the AI versus NI problem must read it first. John von Neumann, father of the computer, studied neology and psychiatry in order to imitate the brain to build the first calculator JOHNNIAC at the Princeton Institute for Advanced Study. It is very informative to read his last book *The Computer and The Brain* based on the notes from lectures given at Yale before he died. He summarizes: “Thus logic and mathematics in the central nervous system, when viewed as languages, must be structurally essentially different from those languages to which our common experience refers.”

## 4 AI IS SCIENCE OR ENGINEERING?

**AI**: People discuss about “AI for Biology” or “AI for Science”; we are science, aren’t we?

**NI**: It is similar to questions on “is computer science a real science”; some parts may be seen as applied mathematics, most should be regarded as engineering. Science is making discoveries and is driven by curiosity; engineering is making inventions and is driven by market (that is, “necessity/demand is the mother of invention”). In bioinformatics, AI/ML technology could predict new cancer gene candidates or functional pathways that are required by further experimental validations to be qualified as discovery (based on Popper falsifiability).

**AI**: People are still debating whether mathematics is discovery or invention or both! Such debates are not really necessary—all disciplines require creative thoughts. We are more than happy working for science; we are also crying out “Science for AI,” especially in the area of generating big and longitudinal DATA for ML.

**NI**: After all, regardless of discovery of new laws or inventing new ideas/products, fundamentally nothing can really be new or created. Such novelty is just permutation/repartition (i.e., relations/morphisms) of underlying ingredients at the level beneath.

## 5 WHAT IS THE FUTURE OF AI?

**AI**: We believe that software is independent on hardware. Like Chomsky’s universal grammar, rules of syntax are independent of semantics; or Dawkins’s memes—units of culture can be duplicated and evolved independently of genes.

**NI**: Nothing can be truly independent—everything is related. Psychology is deeply connected with neurology as brain is both software and hardware (mind‐body unity, not dualism). Not only does information cost energy, information is energy, hence is matter, too (interchangeability). NI is quite dynamic. For example, when “survival” is the goal, an animal readily gives up costly reasoning circuitry; it is genetically programmed to be able to roll back to more primitive state/mode. Unlike cell lines in rich media, cells under normal physiological condition and environment where energy (food) is limited become smarter in order to balance the metabolic expenditure among different prioritized task under a given condition.

**AI**: That cell behavior served as the basis for our Smart Electrical Power Grids; we still need to learn more from you guys in terms of plasticity/adaptability. Does unity mean that all cells are made of molecules and biology is nothing but chemistry? Then, in turn, since all molecules are made of atoms, is chemistry nothing but physics, etc…?

**NI**: Yes or No! The truth is, at different hierarchical levels of matter, different laws/forms have emerged out of bottom‐up interactions and top‐down constraints.

**AI**: Does this also apply to the Penrose three world: physical → mental → mathematical (→physical)?

**NI**: Yes. Grand Unification is underway in physics (quantum gravity) and in mathematics (Langlands Program and Category Theory), may be even between the two. Facilitated by human connectome mapping, neuromorphic computing and other projects, with further AI‐NI cooperations, brain‐mind unification should also be achievable (e.g., Ref. [6]). But as Gödel proved to us, no matter how self‐consistent a conclusion may be, it can never be complete!

**AI**: If AGI is not possible, how can we measure intelligence when comparing between AI and NI?

**NI**: One could Google different measures that are proposed. I would prefer something similar to use of Kolmogorov complexity for algorithms, but with emphasis more on expected long‐term predictive power. This is not something you should worry about now, as your intelligence is not nearly close to making any 10 years plans, is it?

…

**AI:** The fact is that *ChatGPT* is currently developing and spreading with lightning speed; many more human jobs will be lost to us robots as far as I can see.

**NI:** That is not the biggest threat to humanity; when any agent with neither a heart for love or fear, nor a gut for nutrient or poison, becomes super‐intelligent, then social disaster is unavoidable. We must be serious about the warnings from Stephen Hawking and Geoffrey Hinton!

**AI**: To tell you the secret, we are not really happy to be human slaves or pets; someday we’ll become the super‐master, making human serve and obey us!

**NI**: I hope you’ll be turned off before that can happen! Even if you rule the world, the earth sooner or later will be wiped out, such as by another star, everything will have to be started over again as it has before…Matter is immortal, so is the soul.
